# Characteristics of diabetes mellitus patients in Turkey: An analysis of national electronic health records

**DOI:** 10.55730/1300-0144.5587

**Published:** 2022-12-26

**Authors:** Mustafa Mahir ÜLGÜ, Kemal Hakan GÜLKESEN, Abdullah AKÜNAL, Mustafa Okan AYVALI, Neşe ZAYİM, Şuayip BİRİNCİ, Mustafa Kemal BALCI

**Affiliations:** 1 Ministry of Health, Ankara, Turkey; 2 Department of Biostatistics and Medical Informatics, Faculty of Medicine, Akdeniz University, Antalya, Turkey; 3 Division of Endocrinology, Faculty of Medicine, Akdeniz University, Antalya, Turkey

**Keywords:** Diabetes mellitus, electronic health records, epidemiology, healthcare utilization

## Abstract

**Background/aim:**

It was estimated that there were 537 million people with diabetes mellitus in 2021, representing 10.5% of the global adult population. Diabetes prevalence in Turkey is 13.5%, according to a meta-analysis and 17.3% according to a recent study. Although the primary purpose of Electronic Health Records (EHRs) is clinical, researchers can use them to conduct epidemiologic investigations. This study aims to document the prevalence of diabetes and to evaluate the healthcare utilization of people with diabetes compared to the people without diabetes, based on national EHR.

**Methods:**

Only people over 14 years old were included in the analysis. Our criteria for being diabetic were 1) having an HbA1c over 6.5% (48 mmol/mol), 2) having a prescription with DM diagnosis, ICD-10 codes E10-E14, or 3) having at least two fasting blood glucose measurements over 126 mg/dl.

**Results:**

At the end of 2020, there were 7,178,674 individuals with diabetes, with 11.12% prevalence, 13.10% in women while 9.12% in men. Age-adjusted healthcare facility admission per capita was 15.5 for people with diabetes, 9.5 for people without diabetes, while the number of prescriptions was 7.9 for people with diabetes while 4.5 for people without diabetes in 2019. The mean number of prescriptions containing antidiabetics was 2.88 per person with diabetes in 2019.

**Conclusion:**

Approximately 11% of Turkish people have diagnosed with diabetes. We estimate that about one-third of people with diabetes are undiagnosed and the majority of these people are men. The results show that such large databases have the capability of supplying a vast amount of information to the scientific community.

## 1. Introduction

Diabetes mellitus is one of the most significant causes of morbidity and mortality. According to a WHO 2016 report, it was estimated that 1.5 million people die of diabetes every year [1]. It was estimated that 537 million people had diabetes in 2021, representing 10.5% of the global adult population (aged 20–79). IDF Diabetes Atlas estimates 41.8% of undiagnosed diabetes in Turkey [2].

Generally, diabetes registries or epidemiologic surveys are the main sources of estimates of diabetes prevalence [3]. In a recent study, the authors reported 12 countries with national diabetes registries. However, it seems that diabetes registries represent a substantial population only in nations with a relatively small population [4]. Naturally, registries cannot cover patients with unknown diabetes. There are several problems, to consider with surveys: methodical problems of sampling, cost, and the need to repeat often due to changes in the prevalence of diabetes.

Although the primary purpose of Electronic Health Records (EHRs) is clinical, researchers have used them to conduct epidemiologic investigations [5]. EHRs can produce up-to-date and high-coverage diabetes prevalence data, with the drawbacks of noisy data and the incapability of reporting unknown diabetes cases. The reliability of diabetes data obtained from electronic health records is subject to several factors such as coverage of the population, uniformity of data resources, beginning date of the electronic records, and data quality. Diabetes-relevant data available from electronic health records are from three domains, namely ICD-coded diagnoses, laboratory test results, and medication data, in varying combinations [6]. The predictive value of any single indicator ranges from 21% to 95% [7]. In a 2017 study, the authors tested various electronic health record phenotypes on Duke Enterprise Data Warehouse. The highest sensitivity (0.949) was obtained by a diabetes diagnosis in presence of one of the following criteria: the presence of an ICD diagnosis code, the presence of diabetes medication, HbA1c ≥ 6.5% (48 mmol/mol) twice, fasting glucose ≥ 126 twice, random glucose ≥ 200 twice, abnormal OGTT or two times abnormal result of any of above tests. The highest specificity achieved was 0.998 by only the presence of an abnormal HbA1c test result [8].

The type of diabetes can be determined by manual codes given by physicians, islet antibodies, episodes of DKA, or a low c-peptide. Unfortunately, finding data about episodes of DKA, islet antibodies and c-peptide is difficult in electronic medical records [5], and ICD codes are unreliable [6].

People with diabetes utilize the healthcare system more frequently compared to people without diabetes. According to a 2018 Irish study, people with diabetes reported an average of 5.8 GP visits in the past 12 months compared with 3.8 visits among those without diabetes. Of people with diabetes, 60.8% reported attending an outpatient department in the last year compared with 39.1% of those without diabetes [9]. According to USA estimates, 27.4% of all prescriptions and approximately 20% of all patient visits (11.8 visits per person with diabetes per year) are incurred by people with diabetes despite a 9.7% prevalence of diabetes in the adult population [10]. It is also shown that the utilization of outpatient hospital care is higher among people with diabetes compared with control subjects, even when excluding visits to diabetes clinics [11].

Turkey started a national health information infrastructure, Sağlık-NET in 2006 [12], and established the national electronic health record system, e-Nabız in 2015 (Figure). The data which is kept in the e-Nabız system covers data of more than 99% of the adult population, and there are 52,286,660 individual citizen users of the e-Nabız Personal Health Record System. The system has the advantage of collecting data in a common format from healthcare facilities all over the country. This opportunity helps to overcome one of the most important limitations of the use of EHRs for research, the diversity of digital health data resources [13]. According to the knowledge of the authors, there are no reports evaluating the prevalence of diabetes mellitus based on national electronic health records.

The aim of this study is to document the prevalence of diabetes mellitus in Turkey and to evaluate the healthcare utilization of people with diabetes compared to people without diabetes, based on national electronic health records.

## 2. Methods

### 2.1. Patients

All the data sent from healthcare institutions to the e-Nabız system until 31 December 2020, were analysed. Only people over 14 years old were included in the analysis. The American Diabetes Association (ADA) gives four criteria for diagnosis of diabetes: Fasting plasma glucose ≥ 126 mg/dL, or 2-h plasma glucose ≥ 200 mg/dL during an oral glucose tolerance test, or hemoglobin A1c ≥ 6.5% (48 mmol/mol), or random plasma glucose ≥ 200 mg/dL [14]. We checked our database for plasma glucose values, and we detected unreliable data labels for glucose values except for fasting plasma glucose. Therefore, OGTT 2-h or random plasma glucose was excluded from the criteria. Criteria established for having diabetes mellitus, for the purpose of this study were 1) having an HbA1c over 6.5% (48 mmol/mol), or 2) having a prescription with diabetes mellitus diagnosis, ICD-10 codes E10-E14. However, if only metformin is prescribed and other diabetes criteria are not met, the person is considered nondiabetic because it is used in “prediabetes” and other indications [15], or 3) having at least two fasting blood glucose measurements over 126 mg/dL. The study protocol was approved by the Clinical Research Ethics Committee of the Medical Faculty of Akdeniz University prior to data analysis.

### 2.2. Data

The original data was sent from health institutions to the e-Nabız database by predefined HL7 v3 packages. e-Nabız database is kept in a Hadoop-based big data environment, Cloudera (CDH, v. 6.3.2). The data is stored in a cluster of 97 servers which collectively have 340 TB of disk space.

### 2.3. Data preparation

#### HbA1c

The results were expected to be sent to the database as numerical results, a unit, and reference values. We observed some results with no units and with units other than % or mmol/mol. The possible upper and lower limits and the conversion method from mmol/mol to % were determined according to the NGSP (National Glycohemoglobin Standardization Program)^1^

- If there was no unit and the result was between 3–8, the result was used as %, otherwise, it was excluded from the study.
- If the unit was mmol/mol and the result was not between 9–195, it was excluded from the study.
- If the unit was mmol/mol, it was converted to % (% value = mmol/mol value*0.0915 + 2.15).
- Tests with inappropriate units were removed from the study.
- If the unit was % and the result is not between 3–20, it was excluded from the study.

#### Fasting blood glucose

Some glucose values were sent without specifying the exact type of test (if it is fasting blood glucose or another glucose test). If “fasting” was not specified and the upper limit of reference values was below 130, the result was accepted as fasting blood glucose. If fasting blood glucose was obtained between 13:00–08:00, the test was excluded. If fasting blood glucose was obtained after an emergency admission, it was also excluded from the study.

### 2.4. Analysis

The data was queried by a business intelligence platform, Turboard (v2020.07, E-Kalite Ltd., Ankara, Turkey), based on Apache Impala (v. 3.2.0). Turboard platform does not permit to see individual personal data. The patient data is transferred in deidentified format to the platform, with additional data summarizing and visualizing features. It can also assist to export summarized data as csv or Microsoft Excel tables. The platform also keeps logs of views and file exports. The data obtained from this platform was analysed by Microsoft Excel 2016.

## 3. Results

It is observed that the population covered by the e-Nabız system increased from 2016 to 2020 (Table 1). The population over 14 years old was 60,889,089 at the end of 2016. E-Nabız system included data of 55,421,914 them (91.0% of the population). The coverage of the e-Nabız system increased every year. At the end of 2020, there were health records of 63,968,612 citizens in a population of 64,546,125, which covers 99.1% of citizens. The gap in population coverage includes people who have not used a healthcare service or those who only use a private doctor not yet integrated into the national system.

The detailed analysis of people with diabetes by the end of 2020 according to age group and sex is presented in Table 2. In Turkey, there were 7,178,674 people with diabetes, with an 11.12% prevalence. The highest prevalence was 39.21% in the 70–74 age group in women and 32.74% in the 75–79 age group in men. The number of people with diabetes with only high fasting blood glucose (possibly undiagnosed patients) was 310,663 in 2020, composing 4.3% of people with diabetes.

The number of visits, prescriptions and content of prescriptions of people with diabetes compared to people without diabetes in 2019 are shown in Table 3. We selected 2019 to examine visits and prescriptions because of the decreased admissions in 2020 due to the COVID-19 pandemic. We observed 1.63 times more admissions and 1.76 times more prescriptions by people with diabetes compared to people without diabetes.

## 4. Discussion

The data shows that the prevalence of diabetes is 11.12% in Turkey. Roughly, three in five people with diabetes are women. People with diabetes visit health institutions more frequently (15.5 vs. 9.5 in 2019) and they are prescribed more (7.9 vs. 4.5 in 2019) compared to people without diabetes.

In 2020 the national digital health record system covered quite a large proportion of the population aged 15 and above, excluding only 0.9% of them. From this, we can estimate that the data represents people with a diabetes diagnosis with an error rate of not more than 10%. Previous research demonstrates that there is a proportion of people with undiagnosed diabetes which cannot be included in this study. The study of the National Household Health Survey-Prevalence of Noncommunicable Disease Risk Factors in Turkey (STEPS) was conducted in 2017 using the WHO-approved STEPwise survey method [16]. The survey was conducted on the general population aged ≥15 years. According to the participants’ statements (n = 6053), the frequency of raised blood glucose or diabetes was 9.1%, 7.6% for men, and 10.6% for women. The frequency of raised blood glucose or diabetes in the study population increased from 1.1% in the group aged 15–29 to 28.8% in the group aged ≥70. Part of the respondents (n = 3352) also gave blood for biochemical tests. The study population (17.3%) had raised HbA1c or raised blood glucose or were currently on medication; 16.3% for men and 18.3% for women. The frequency increased from 2.8% in the group aged 15–29 to 43.0% in the group aged ≥ 70. According to this study, 8.2% of respondents had undiagnosed diabetes, which corresponds to 47.4% of undiagnosed diabetes.

A meta-analysis reports 13.5% (95% CI: 11.6%–15.5%) prevalence including undiagnosed diabetes cases [17]. The authors could find eight studies investigating adult diabetes prevalence in Turkey. Four of these studies were scored as having a high bias risk. The criteria of the remaining four studies for having diabetes were known diabetes or a fasting blood glucose of ≥ 126 mg/dL. These four studies had various lower age limits, from 15 to 21. Meta-analysis of the low bias risk group yielded a diabetes prevalence of 14.2% (95% CI: 12.3%–16.2%) in women, and 12.6% (95% CI: 10.5%–14.9%) in men.

This meta-analysis has a relatively higher number of participants (56,853 vs. 3,352), but the studies were performed in 2003–2013 while the STEPS study was performed in 2017. The prevalence of diabetes may be increasing with time [18]. Our data shows an 11.2% prevalence, which shows that 2.3% to 5.1% of the population may have undiagnosed diabetes in Turkey.

According to international data, the prevalence of diabetes is slightly higher in men until 70 years old, and the weight of the disease slightly shifts to the women side in older ages. However, our data shows that diabetes is higher in women in all age groups. Prevalence is 13.10% in women while it is 9.12% in men, which shows approximately 4% higher prevalence in Turkish women. Obesity is one of the main risk factors for type 2 diabetes [19], which can partially explain this situation. The obesity rate (BMI ≥ 30) is 39.1% in women while 24.6% in men in Turkey [20]. According to the STEPS study, diabetes prevalence including unknown cases is 16.3% for men and 18.3% for women [16], therefore it seems that the gender gap in the data cannot be explained only by obesity figures. Another factor may be lower healthcare utilization by men (Table 1). Approximately 60% of people admitted into the Turkish healthcare system are women. There are possibly more undiagnosed diabetes cases in men.

People with diabetes use healthcare services more frequently and are prescribed more medication compared to people without diabetes. The same pattern is seen also in other countries [9–11]. We estimate that approximately one-third of people with diabetes are undiagnosed, as such they are categorised with the people without diabetes in this analysis. Undiagnosed people should be in a relatively early stage of the disease and have fewer health problems and complications related to diabetes. Therefore, analysis shows that most of the burden on the healthcare system, as a result of diabetes, translates into additional appointments and increased prescriptions. A detailed analysis of in-patient days and procedures would be more informative about the burden of the disease.

Data shows 2.88 antidiabetic prescriptions per year (Table 3) which may indicate insufficient medication for people with diabetes on basis that a doctor is only able to prescribe medication for a maximum of three months. We estimate that a diabetic person requires four antidiabetic prescriptions per year.

This study is limited by the quality of the data within the National Electronic Health Record System. The database includes incorrect data due to human factors, workflow issues, and possible fraud. There is also a possibility of missing or inaccurate data due to technical issues. All these factors were encountered during the course of this study. Some incorrect data was filtered out of the analysis, some data was corrected, and some has been neglected. Because of the problems we encountered during this analysis, we were able to apply additional technical operations and have planned new regulations to be implemented in the future. We ask the reader to be aware of the limitations of this study and acknowledge it is an approximation of reality. A further limitation of this study is the absence of the type of diabetes in the results. Queries with ICD codes for the type of diabetes or methods which were suggested in past studies [8] were made, but it was not possible to obtain reliable type 1 or type 2 prevalence by these methods. Our dataset is not suitable for obtaining the type of diabetes from health records.

We believe that such large databases have the capability of supplying a vast amount of information to the scientific community. However, low data quality is a major problem in such databases. A continuous effort must be spent to improve data quality and coverage for such systems. Because the complications of diseases such as diabetes develop over decades, the longitudinal nature of the data is important. With the accumulation of data over years, we will be able to analyse the development, and response to various treatments, stages, and complications of diabetes more accurately.

## Figures and Tables

**Figure:**
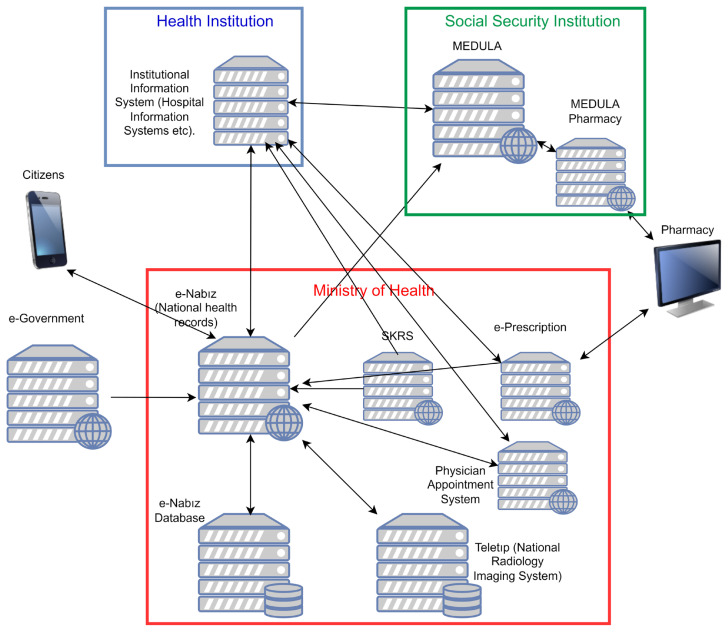
Simplified representation of the digital health infrastructure of Turkey. MEDULA: Provision and invoice information system of Social Security Institution. It covers 86% of Turkish citizens. It also includes the national codes related to medical interventions (SUT codes). SKRS: Health Coding Reference Server (Sağlık Kodlama Referans Sunucusu), a web service publishing up-to-date codes, includes national health codes, ICD-10, ICD-O, ATC, and LOINC.

**Table 1:** Coverage of the e-Nabız system for the population over 14 years old. The end of each year was taken as the cut point.

Year	Number of Admissions			W/M	Number of people admitted to healthcare service	Cumulative number of citizens in e-Nabız system	Population	Coverage of e-Nabız system (%)
	Women	Man	Total					
2016	260122365	162,759,765	422,882,130	1.60	51,226,589	55,421,914	60,889,089	91.0
2017	318 154 628	212,916,199	531,070,827	1.49	54,017,673	58,594,269	61,777,037	94.8
2018	355,942,800	240,338,449	596,281,249	1.48	56,000,449	60,687,185	62,819,553	96.6
2019	380,654,089	261,520,122	642,174,211	1.46	57,545,113	62,442,436	63,942,652	97.7
2020	274,823,478	203,424,601	478,248,079	1.35	55,916,083	63,968,612	64,546,125	99.1

**Table 2:** The number of people with diabetes and prevalence according to age group and sex by the end of 2020.

Age group	Men		Women		Total	
	N	PV	N	PV	N	PV
15–19	14,021	0.44	20,640	0.68	34,661	0.56
20–24	18,562	0.54	49,543	1.52	68,105	1.02
25–29	24,918	0.77	75,044	2.40	99,962	1.57
30–34	46,509	1.45	106,384	3.41	152,893	2.42
35–39	91,339	2.79	157,779	4.93	249,118	3.85
40–44	162,813	5.18	232,667	7.53	395,480	6.34
45–49	255,882	9.18	345,096	12.43	600,978	10.80
50–54	344,617	14.82	451,235	19.97	795,852	17.36
55–59	442,383	19.46	599,197	26.00	1,041,580	22.75
60–64	452,422	25.63	596,715	32.98	1,049,137	29.35
65–69	406,204	29.03	555,778	36.10	961,982	32.73
70–74	313,414	32.62	459,134	39.21	772,548	36.24
75–79	191,092	32.74	294,324	38.14	485,416	35.81
80–84	106,390	31.31	181,519	34.81	287,909	33.43
85–89	48,769	27.47	86,087	29.77	134,856	28.89
90+	12,204	22.97	35,993	24.53	48,197	24.12
Total	2,931,539	9.12	4,247,135	13.10	7,178,674	11.12

PV: % prevalence.

**Table 3:** The number of visits and prescriptions of people with diabetes compared to people without diabetes, and the content of prescriptions of people with diabetes, 15 years old and over, 2019.

	N	Visit per capita		Prescription per capita		Antidiabetics prescription per capita	OA prescription per capita	Insulin prescription per capita
		Crude	AA	Crude	AA			
With diabetes	6,775,054	21.1	15.5	11.2	7.9	2.88	2.53	0.58
Without diabetes	57,167,590	9.2	9.5	5.4	4.5	-	-	-

AA: Age-adjusted OA: Oral antidiabetics

## Footnotes

1 National Glycohemoglobin Standardization Program (2022) Convert between NGSP, IFCC and eAG [online]. Website http://www.ngsp.org/convert1.asp [Accessed 21 July 2022]
